# Secretory RING finger proteins function as effectors in a grapevine galling insect

**DOI:** 10.1186/s12864-019-6313-x

**Published:** 2019-12-03

**Authors:** Chaoyang Zhao, Claude Rispe, Paul D. Nabity

**Affiliations:** 1 Department of Botany and Plant Sciences, University of California, Riverside, CA USA; 2 BIOEPAR, INRA, Oniris, Nantes, France

**Keywords:** E3 ligase, Gall formation, Grape phylloxera, Herbivore, Proteasome

## Abstract

**Background:**

All eukaryotes share a conserved network of processes regulated by the proteasome and fundamental to growth, development, or perception of the environment, leading to complex but often predictable responses to stress. As a specialized component of the ubiquitin-proteasome system (UPS), the RING finger domain mediates protein-protein interactions and displays considerable versatility in regulating many physiological processes in plants. Many pathogenic organisms co-opt the UPS through RING-type E3 ligases, but little is known about how insects modify these integral networks to generate novel plant phenotypes.

**Results:**

Using a combination of transcriptome sequencing and genome annotation of a grapevine galling species, *Daktulosphaira vitifoliae*, we identified 138 putatively secretory protein RING-type (SPRINGs) E3 ligases that showed structure and evolutionary signatures of genes under rapid evolution. Moreover, the majority of the SPRINGs were more expressed in the feeding stage than the non-feeding egg stage, in contrast to the non-secretory RING genes. Phylogenetic analyses indicated that the SPRINGs formed clusters, likely resulting from species-specific gene duplication and conforming to features of arthropod host-manipulating (effector) genes. To test the hypothesis that these SPRINGs evolved to manipulate cellular processes within the plant host, we examined SPRING interactions with grapevine proteins using the yeast two-hybrid assay. An insect SPRING interacted with two plant proteins, a cellulose synthase, CSLD5, and a ribosomal protein, RPS4B suggesting secretion reprograms host immune signaling, cell division, and stress response in favor of the insect. Plant UPS gene expression during gall development linked numerous processes to novel organogenesis.

**Conclusions:**

Taken together, *D. vitifoliae* SPRINGs represent a novel gene expansion that evolved to interact with *Vitis* hosts. Thus, a pattern is emerging for gall forming insects to manipulate plant development through UPS targeting.

## Background

The ubiquitin-proteasome system (UPS) is a major protein turnover pathway found across the domains of life, but is especially important in regulating almost all plant development and signaling pathways. Hormone-driven growth, ontogenetic change, and responses to stress represent key cellular processes controlled by ubiquitin mediated degradation [1–3]. Selective protein turnover is considered advantageous largely because it reduces the time to enact or cease metabolic processes and also prevent inappropriate reactivation [4]; however, these exact traits, in addition to the conservation in function and ubiquitous nature, create a strong selective environment to target the UPS as a mode of plant manipulation by other organisms. Indeed, all manner of plant antagonists evolved ways to alter plant-host UPS processes. Numerous reviews highlight what component processes of the UPS serve as targets for pathogens (e.g., [5, 6]) , [7, 8], fungi [9], and nematodes [10] mimic E3 ligases to compromise immunity and promote colonization. Although less is known about insects, evidence is emerging that salivary secretions contain proteasome modifying enzymes [11, 12], indicating convergent strategies among plant manipulating eukaryotes.

Within the UPS, most E3 ubiquitin ligases function to recognize both E2 domains and additional domains of a target substrate to facilitate the transfer of ubiquitin, thus promoting protein turnover and stabilization. E3 genes typically make up the largest gene families in plants with elevated rates of evolution, reflecting their broad regulation of specific substrates, and also show enrichment in intronless genes, an artifact of the evolution of genes that function in rapid turnover [13–15]. Three gene families make up all E3 ligases, including proteins with homology to E6AP C-terminus or HECT proteins, U-box proteins, and RING-domain E3 ligases (RING E3s), of which the RING genes comprise the largest family [16], and subsequently play myriad roles in cellular and organelle regulation including plant immune and defense function [17]. For example, RING containing genes interact with fungal and bacterial effectors to regulate hormone-mediated defense pathways [18, 19].

E3 ligases also hone plant defense against insect antagonists during feeding or attempted colonization. For insects that induce jasmonate (JA)-related defenses, JAZ repressor proteins interact with the Skp1/Cullin1/F-box (SCFCOI1) E3 complex to transfer ubiquitin and degrade the select JAZ protein in the proteasome, thereby activating JA defense [20]. JA is also repressed by associated genes, such as JAV1, that require proteasomal degradation to allow for defense signaling [21]. Similarly, the initiation of salicylic acid (SA) defenses are regulated through the proteasomal degradation of transcription factors (e.g., WRKY45) and regulatory proteins (e.g., non-expressor of pathogen related gene 1; NPR1) where constitutive expression is suppressed to prevent unnecessary SA signaling until an attack occurs [22, 23]. Given the role of E3 ligases in controlling plant defense signaling and the ubiquitous nature of E3 ligases among organisms, there is likely increased selection among plant-insect interactions for insects to evolve proteins to hijack plant E3 function and deactivate plant defenses.

Although the function of all predicted insect effectors is unresolved, evidence suggests they 1) deactivate plant defenses or innate immune signaling, thus enabling colonization and sustained feeding [24–27], and likely 2) augment growth and/or development to enhance nutritional gains [28, 29]. For some highly specialized insects that initiate hyperplasia or novel organ growth called galls, the induced phenotype is extraordinarily complex, encompassing well defined cellular layers, localized defense and nutritive compounds, and novel morphology synthesis [30–33]. How these phenotypes arise mechanistically remains unknown; however, the dominant hypotheses include effector-driven initiation and maintenance of galls [12, 34, 35] and hormone induced tissue differentiation [36]. Comparative analyses among congeneric gallers [37] correlates genes encoding secretory proteins to their life history, but additional examinations are required to understand the evolutionary origin, significance, and functions of these types of proteins in search of evidence in support of existing hypotheses. To this end, we combined transcriptome and genome sequencing with protein interaction assays to characterize a discrete clade of genes with effector-like attributes in the cosmopolitan and agriculturally significant galling herbivore, *Daktulosphaira vitifoliae*.

Grape phylloxera, *D. vitifoliae*, is a pest of cultivated grapevine, with a nearly worldwide distribution, following its accidental introduction from Northern America to the rest of the world. In its native range, *D. vitifoliae* feeds on several species in the *Vitis* genus, on which it forms galls (on leaves and roots) but does not appear to cause significant damage. By contrast, the introduction of *D. vitifoliae* to the Old World wreaked havoc on the culture of the cultivated vine, *Vitis vinifera*. This difference likely results from the coevolution of this insect with different host species within the native range, whereas cultivated vines had not been exposed to the insect and remained highly sensitive. Global vine production was in fact brought to a near-collapse until the discovery that grafting on resistant wild American *Vitis* provided resistance. Despite the economic significance of grapevines, and the fact that this biological invasion dates back to the mid-nineteenth century and generated considerable research, how the grape-phylloxera-insect initiates and sustains gall formation remains unknown. Thus, we attempt to fill this knowledge gap by bringing insight on the mechanisms used by phylloxera to manipulate its host plants.

## Results

### *D. vitifoliae* encodes a large number of secretory RING finger protein genes

**Fig. 1:**
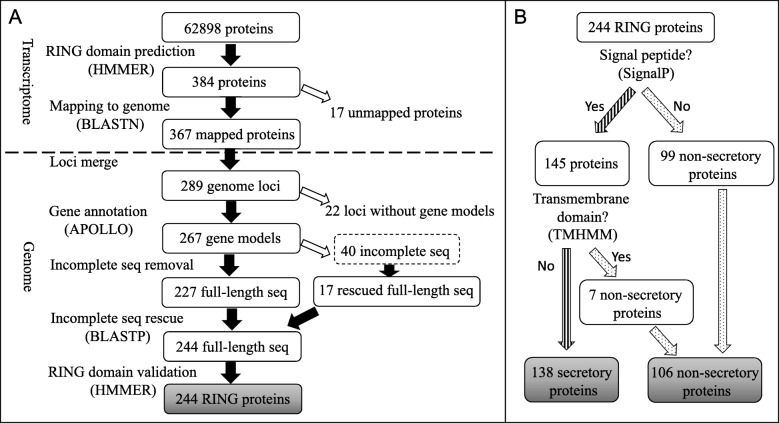
Bioinformatics pipeline to identify putatively secretory and non-secretory RING finger proteins from *D. vitifoliae*. **a** Screening of full-length RING finger proteins using the bioinformatics tools. **b** Selection of secretory and non-secretory RING finger proteins

In this study, we developed a bioinformatics pipeline that incorporated both transcriptome and genome sequences to predict non-secretory RING finger proteins and SPRINGs in *D. vitifoliae* (Fig. 1). From the 62,898 transcriptome-derived protein sequences that were used to screen against the Pfam domain database, 384 hit CL0229, a RING clan comprising 43 families of RING zinc finger domains and the U-box domain [49]. Because the alignments of 17 protein hits to the genome sequence fell below a 90% identity threshold, only the remaining 367 were used to further collapse into 289 genome loci, among which 22 were disregarded due to the unavailability of gene models. From the remaining 267 annotated gene models, 227 were determined to be full-length, while sequence gaps of another 17 were filled using the transcriptome sequences, giving rise to a total of 244 full-length sequences whose RING domains were validated through HMMERSCAN searches (Fig. 1a). Among these, 138 were predicted as SPRINGs for the presence of signal peptides and absence of transmembrane domains, and the other 106 were predicted as non-secretory RING proteins for lacking signal peptides or containing transmembrane domains (Fig. 1b; Additional file 1).

### SPRINGs were small-sized and evolutionarily non-conserved, relative to the non-secretory proteins

**Fig. 2:**
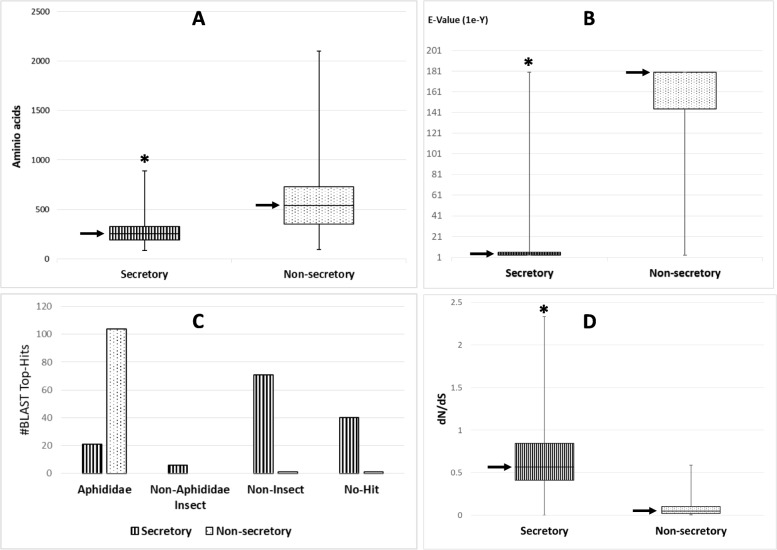
Comparison of secretory and non-secretory RING proteins on protein length (**a**), BLAST top hit E-values (**b**), BLAST top hit species (**c**), and dN/dS ratio (**d**). Positions of the box plot medians are indicated by arrows, and significant differences between the secretory and non-secretory RING protein groups are indicated by asterisks (unpaired t-test *p*-value < 0.01)

Comparisons of SPRINGs and non-secretory RING proteins revealed that SPRINGs (median size = 252 aa) were significantly smaller (one-tailed unpaired t-test *p*-value < 0.01) than the non-secretory proteins (median size = 538 aa; Fig. 2a). Sequence homology searches (BLASTP) indicated that the secretory RING proteins mostly showed little to no sequence similarity to the known proteins deposited in the GenBank databases, in contrast to the non-secretory proteins, most of which were highly similar to known proteins. The median top hit e-value was 1e-4 for secretory proteins and 1e-180 for non-secretory proteins (Fig. 2b). In addition, compared to the non-secretory RING proteins that were most similar to their Aphididae homologs (104 top BLAST hit species are aphids), the secretory RING proteins were mostly similar to non-insect species (71 top hits), or specific to *D. vitifoliae* (40 top hits) based on an e-value = 1e-3 threshold (Fig. 2c). Overall, thus, this shows a stark contrast between non-secretory RINGs, which almost always have homologs in aphid, with high conservation of sequences, and secretory RINGs which are often no-hit or at least have very low levels of sequence conservation. Molecular rate analysis of gene families within secretory and non-secretory RINGs also showed that the nonsynonymous to synonymous substitution rate ratio (dN/dS) was significantly higher (one-tailed unpaired t-test *p*-value < 0.01) in the secretory proteins (median = 0.57) than in the non-secretory proteins (median = 0.05). This demonstrates that the former evolved rapidly, either under relaxed or positive selection (Fig. 2d), while the latter evolves under purifying selection.

### Secretory RING protein genes are more likely to duplicate and express at feeding stage

**Fig. 3:**
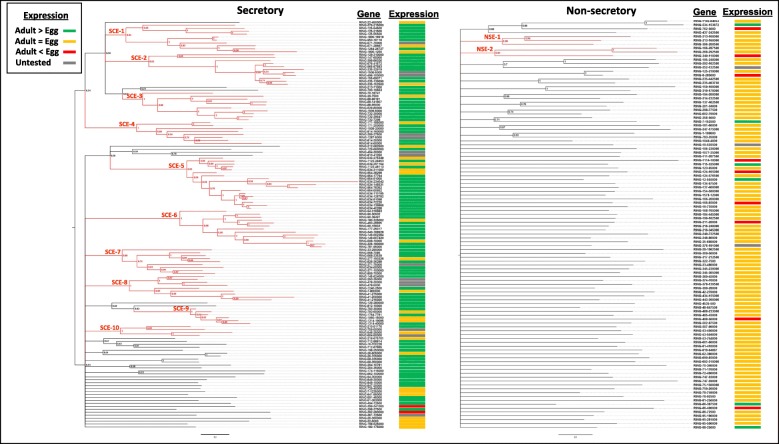
Phylogenies and expression profiles of secretory (left) and non-secretory RING proteins. Clades comprising ≥3 members and supported with ≥0.9 posterior probabilities were highlighted in red. Feeding Adult (*n* = 4) were compared against nonfeeding Egg (*n* = 3) stages

To understand how the RING proteins evolved in *D. vitifoliae*, we constructed the Bayesian inference of phylogenetic trees separately for secretory and no-secretory RING proteins because of the sequence divergence between these two groups. Based on a 0.9 posterior probabilities threshold to cluster ≥3 RING proteins, only six (6%) non-secretory proteins formed two clusters, NSE-1 and NSE-2 (Fig. 3). Even the clustering thresholds were lowered to 0.5 posterior probabilities for ≥2 RING proteins, the majority (75, or 71%) of the non-secretory RING proteins still existed as singletons. However, in the secretory group, 98 (71%) RING proteins formed 10 clusters (SCE-1 through SCE-10) with the largest including 18 members, based on the stringent clustering threshold used above (Fig. 3). When using the loose threshold, 120 (87%) of secretory RING proteins formed clusters. In addition, we observed that clusters in the secretory RING group were often formed by genes adjacent in the genome sequence, for example, 11 SCE-5 genes were located in scaffold #534, suggesting a pattern of recurrent tandem duplication that caused the expansion of secretory RING genes in *D. vitifoliae* (Fig. 3). Given that most of these secretory RING proteins showed little to no sequence similarity to other known proteins, they are likely to have multiplied in the insect genome through species-specific gene duplication.

RNA-Seq-based expression analyses indicated that the secretory and non-secretory RING protein genes displayed strong expression profiles distinct to each insect life stage and fewer genes specific to location. Out of the 124 tested secretory protein genes, 94 (75%) were higher expressed in adults than in eggs, and only 2 were higher expressed in eggs than in adults (Fig. 3). In contrast, only 6 (6%), out of 103 tested non-secretory protein genes were higher expressed in adults than in eggs, and the majority (88, or 85%) were similarly expressed in both life stages. Notably, expression of 9 (8%) non-secretory protein genes were even upregulated in eggs in relative to adults (Fig. 3). Of these genes 16 secretory and 3 non-secretory were expressed higher in North America origin individuals whereas 6 genes of each type were expressed higher in France origin individuals (Additional file 1). Eggshell related genes were largely downregulated in adult tissues, and mitochondria specific genes were constitutively expressed across tissues, indicating expression profiles represent valid comparisons. Thus, the patterns of expression by both secretory and non-secretory genes are not driven by genetic differences among populations and the secretory ones are more likely to function during insect feeding.

### The ring domain interacts with host proteins

**Fig. 4:**
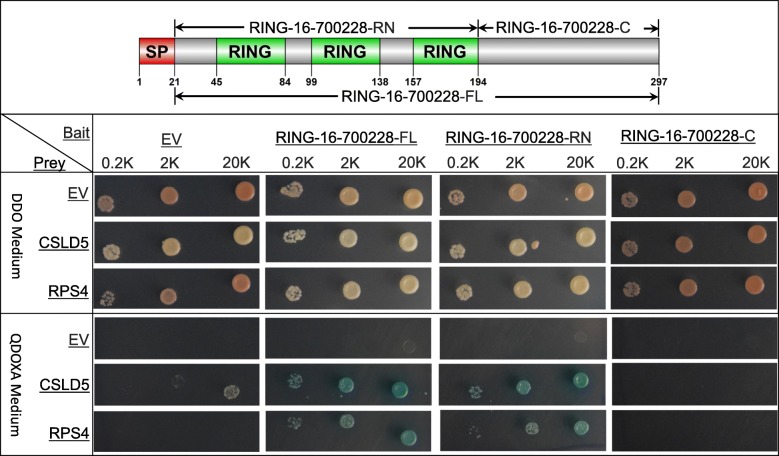
Pairwise yeast two-hybrid assay of protein-protein interactions between a *D. vitifoliae* RING protein (RING-16-700228) and two *V. vinifera* proteins (CSLD5 and RPS4B). Top: Three fragments of the bait RING protein, the full-length (RING-16-700228-FL) with the signal peptide (SP) domain removed, the RING domain (RING-16-700228-RN), and the C-terminus (RING-16-700228-C), were respectively cloned as baits for the Y2H assay. Bottom: Transformed yeast clones containing both bait and prey plasmids were tested in three dilutions, 200 (0.2 K), 2000 (2 K), and 20,000 (20 K) cells per drop, on DDO and QDOXA medium, respectively. Empty vectors (EV) were used as negative control

To investigate whether the secretory RING proteins are injected by insects during their feeding for host manipulation, we selected a secretory protein, RING-16-700228, whose expression was upregulated in the feeding stage compared to the non-feeding stage, as the bait to test its interactions with plant proteins using the yeast two-hybrid assay. The full-length coding sequence, excluding the signal peptide-encoding region, was constructed in a bait clone to screen the *V. vinifera* leaf prey library using yeast mating. After eliminating duplicates, only two *V. vinifera* prey proteins, the cellulose synthase-like protein D5 (CSLD5, Gene ID # 100243459) and 40S ribosomal protein S4–1 (RPS4, Gene ID # 100244922), were found to interact with the bait RING protein. We then performed pairwise yeast two-hybrid analysis by co-transforming the bait and prey plasmids in pairs into the Y2HGold yeast cells, and tested their interactions using the high-stringency selective medium QDOXA. Only the presence of both plant (CSLD5 or RPS4) and insect (RING-16-700228) proteins in same yeast cells was able to activate the reporter genes, while the plant or insect proteins alone was not (Fig. 4), indicating that the *D. vitifoliae* RING protein interacted with both CSLD5 and RPS4 proteins. To determine whether the RING domain, which is composed of three RING fingers, or the C-terminal fragment without RING finger, of RING-16-700228 interacted with the plant proteins, both fragments were cloned for the pairwise yeast two-hybrid assay (Fig. 4). Similar as the full-length protein, the RING domain fragment, but not the C-terminal fragment, interacted with both plant CSLD5 and RPS4 proteins, indicating that the RING fingers are the target-binding domain in RING-16-700228.

### Proteasomal function and host targets are upregulated during gall formation

Transcriptome profiling of native *D. vitifoliae*-*V. riparia* interactions revealed less expression in plant genes assigned proteasomal function for flower, bud, and gall development relative to leaf expression than expected and no enrichment at each stage of gall development or for leaf development (Additional file 1). Among differentially expressed genes, galled tissues showed greater numbers of downregulated proteasomal genes across all stages, and this was linked to a strong suppression in protein synthesis-related genes. Select genes identified as linking reproductive pathway activity to gall tissues were expressed similar as in the original study [47] in galled vs non-galled tissues (Additional file 1). Blasts of the *V. vinifera* genome identified the protein targets from the above interaction assays as GSVIVT01028071001 (CSLD5) and GSVIVT01032756001 (RPS4B). For CSLD5, gall tissue showed higher expression than leaves and reduced expression during leaf development or when comparing flowers to leaves. Bud tissue showed slightly elevated expression compared to leaves (Table 1). Expression for RPS4B was elevated in gall tissue compared to leaves and reduced during leaf development, whereas flower or bud tissue compared to leaves showed reduced expression or no change (Table 1).

**Table 1:** Expression profiles of *V. vinifera* genes identified as targets of *D. vitifoliae* SPRING (RING-16-700228) for different tissue types

Contrasts:	Gall-Leaf	Leaf Develop.	Flower-Leaf	Bud-Leaf	Gall1	Gall2	Gall3	Gall4
RPS4B: GSVIVT01032756001								
Log FC	1.54	−1.60	−0.49	0.27	1.21	1.02	1.14	1.99
Adj. *P* value	2.32E-12	9.05E-08	0.002	0.096	1.12E-05	5.83E-05	1.14E-05	1.73E-09
CSLD5: GSVIVT01028071001								
Log FC	0.62	−5.44	−2.08	1.17	−0.65	−0.93	−0.94	2.91
Adj. *P* value	0.001	2.57E-12	4.62E-08	0.0001	0.077	0.012	0.009	1.18E-08

## Discussion

During the genome and transcriptome curation of the gall inducing *D. vitifoliae* we identified a large cluster of related genes that encode RING proteins and retain secretory characteristics, suggesting a role in host protein-protein interactions. Previous studies characterize arthropod effectors as 1) secretory, 2) small-sized, 3) fast-evolving, 4) lineage-specific, 5) cluster-forming, and 6) feeding related [50, 51]. In contrast to the *D. vitifoliae* non-secretory RING proteins, the SPRINGs are secretory, smaller (median length of 252 aa), exhibit high dN/dS values, a signature of rapid evolution common among effectors, and show lineage specific duplications of genes that cluster together. Thus *D. vitifoliae* SPRINGs share characteristics of effectors and likely retain duplicate function or duplicate structures with neofunctionalization to modify plants [52–58]. Our gene expression analysis across life stages provided further support that *D. vitifoliae* SPRINGs may modify plants because most SPRINGs showed upregulation during the feeding stages. In contrast, the non-secretory RING proteins showed similar expression between the feeding adult and non-feeding egg stages with some even more expressed in eggs than adults, linking the non-secretory RING proteins to insect homeostasis or development. Finally, our interaction assays identified possible host proteins targeted by SPRINGs, confirming the insect proteins bind to plant proteins. Altogether, these results provide support for our hypothesis that the SPRINGs function as effectors to modify *Vitis* hosts.

In eukaryotes, RING proteins are largely present as intracellular molecules within nuclear, mitochondrial, and other subcellular compartments, while others function as transmembrane proteins in the cellular membrane [17]. Among insects, *Drosophila melanogaster* retains 139 RING proteins but all lack secretory function [59], and recent profiling of the plant feeding white-backed planthopper, *Sogatella furcifera*, revealed three RING-related proteins present in salivary secretions [60]: one protein shared a domain similar to the RING type, E3 ubiquitin-protein ligase UBR4, a ubiquitous gene that performs neurological functions in insects [61] and auxin transport in plants [62], and two other unspecified genes contained RING finger domains. Although none of *S. furcifera* proteins showed association with secretory pathways or signal peptides, that the proteins were detected in secretions suggests RING domains mediate host-plant interactions. As more insect herbivore secretomes become available, the RING domain may arise as a convergent strategy used by disparate lineages to influence plant function, and more SPRINGs may be described. In the only other known example, an intracellular protozoan uses a SPRING to manipulate animal host immune function through promiscuous interactions with numerous proteins [43], suggesting a pattern is emerging across kingdoms for SPRINGs to evolve during specialized parasite-host interactions.

RING finger proteins exhibit E3 ubiquitin ligase activity by bringing E2 ubiquitin-conjugating enzyme and a target substrate in close proximity, which catalyzes the transfer of ubiquitin to substrate [63]. This process relies on the interaction between the RING domain and the E2 enzyme [64]. Using a *D. vitifoliae* SPRING, RING-16-700228, to screen its interacting proteins from a *V. vinifera* leaf cDNA library, surprisingly we did not identify E2 enzymes as targets. This may result from false negatives common with Y2H screens [65]; however, an alternative hypothesis is that this RING protein interferes with the recognition or activity of its substrates without manipulating E2 enzymes. Nevertheless, our screening revealed two candidate plant proteins, a cellulose synthase-like protein (CSLD5) and a ribosomal protein (RPS4) that bind to the RING domain of the *D. vitifoliae* SPRING. CSLD5 is required for plant cell division and rapidly degrades after cell division through interaction with an anaphase promoting complex (APC) E3 ligase. Interestingly, the master regulator of stomatal development, SPEECHLESS, binds to the 5′ regulatory region of CSLD5 to facilitate expression while suppressing ROS [66], and reduced expression can lead to aberrant stomatal patterning [67, 68]. Given that *D. vitifoliae* create adaxial stomata where normal leaves lack stomata [33], and gall tissues show aberrant stomatal patterning [48] the regulation of CSLD5 may represent an adaptive target. Gene expression analyses of the ontogeny of gall development on *V. riparia* revealed CSLD5 expression decreased with leaf development, but remained elevated in galls compared to leaves. This suggests enhanced expression of CSLD5 is maintained by *D. vitifoliae* perhaps to regulate ROS, initiate stomatal development, maintain cell division or any combination of these specific to the gall stage, although how SPRING binding to CSLD5 regulates this remains unknown. Perhaps SPRING binding reduces plant self-recognition of CSLD5 thereby leading to reduced turnover through the proteasome, or enhanced expression results from a disruption in feedback-mediated signaling.

The second candidate target, RPS4, likely represents the *V. vinifera* ribosomal protein RPS4B involved in RNA binding. RPS4B is a disease resistance R protein targeted specifically by the *Pseudomonas* avirulence effector (AvrRps4) where interaction triggers a hypersensitive response [69]. Gene expression of RPS4B showed elevated expression in gall versus leaf tissue and reduced expression as leaves develop, suggesting *D. vitifoliae* induces expression, perhaps as a plant defense response. Given RPS4 genes largely influence pathogen resistance [70], *D. vitifoliae* mediated induction suggests an alternative hypothesis that induced RPS4B functions as an extended phenotype to protect the plant and its obligate specialist herbivore. How SPRING binding increases expression is unknown, although a pattern is emerging to suggest positive interactions enhance expression in ways consistent with an adaptive induced phenotype.

Many effectors from distantly related plant antagonists share structural and functional similarities, e.g., protein domains and motifs, demonstrating a fascinating phenomenon of convergent evolution [12, 71]. Among these are plant UPS-targeting effectors that function through mimicking or directly interacting with host UPS components [9, 12, 72–74]. Thus, it is reasonable to hypothesize that the secretory RING proteins identified in this study are effectors of *D. vitifoliae* that engage in protein turnover. In support of this hypothesis we found strong suppression in genes related to protein synthesis with 4–5-fold more genes downregulated in galls than during leaf, flower, or bud development. Given the numerous effectors likely to occur in the *D. vitifoliae* genome and the complexity of organogenesis, modifications to protein synthesis through UPS targeting is not surprising. Additional study of how components of the UPS change relative to the genetic background (effector repertoire) of *D. vitifoliae*, in addition to more functional interaction assays and comparisons with other galling insects will help resolve conserved immune or developmental targets converged upon through adaptive specialization.

## Conclusions

Through comparative genomic and transcriptomic sequencing of a galling herbivore, we characterized an expansion in RING-containing E3 ligases that revealed the accelerated evolution of secretory function, life-stage specific gene expression conducive to manipulating the plant host, and interactions with immune signaling, cell division, and UPS modulation during novel organ development. These SPRINGs represent a second novel evolution of secretory RING function, suggesting convergence in host manipulation across kingdoms. Taken together, these data strongly suggest that *D. vitifoliae* manipulates the UPS to evade or more likely hone host defense and immune signaling during construction of a complex, yet adaptive phenotype. Although limited knowledge exists on galling insect effectors (but see also [12]), a pattern is emerging that gall formation results from host interactions with insect genes within expanded clades of UPS targeting genes, ultimately using protein-interactions to induce the complete gall phenotype.

## Methods

All bioinformatics tools used here were run at default setting unless explicitly stated.

### Insect RNA sequencing and de novo transcriptome assembly

To identify and better understand genes active during feeding, *D. vitifoliae* samples at all life stages were collected from leaves of *V. riparia* host plants from La Crescent, Minnesota (43.885364, 91.338169) and Memphis TN (35.155087, 90.058010). Leaf galls (these are then named ‘gallicoles’ in phylloxera jargon, whereas the ‘radicicole’ form feeds on root nodosities) were dissected and insects removed prior to processing, and eggs and feeding stages were separated. Feeding individuals from MN were used to generate a new transcriptome to use with the genome derived from nonnative European clones. Feeding and non-feeding (egg) stages from TN were used to compare with previously published transcriptomes from European populations [38]. In both cases the goal was to increase genetic diversity using wild native populations to minimize potential genetic bottlenecks associated with using clones from nonnative European populations [38]. All insects were stored in RNAlater solution (Qiagen) at room temperature initially, transferred to 4 °C within 8 h for temporary storage for less than 7 days, and later kept at − 80 °C until RNA isolation. Three replicates of adult insect samples from TN and two replicates of egg samples from TN and of feeding stage from MN, each containing 20–50 juvenile and adult individuals or > 200 eggs, were processed for total RNA extraction using RNeasy Mini kit (Qiagen) according to the manufacturer’s instructions. The mRNA library construction, paired-end Illumina HiSeq RNA sequencing, and de novo transcriptome assembly were performed following the procedures described by [39]. Raw reads have been uploaded to GenBank with accession numbers PRJNA552348 and PRJNA561603.

### Annotation of RING finger protein genes

The longest open reading frames (ORFs) for all transcripts of the *D. vitifoliae* RNA-Seq assembly were predicted and translated into protein sequences using the Blast2GO “Translate Longest ORF” tool. Translated protein sequences were searched against the Pfam domain database (Pfam29.0) using the HMMSCAN program included in the HMMER software suite (version 3.1b1 [40]) for the RING clan (CL0229) which includes RING zinc finger domains and the U-box domain. Transcripts were aligned to the *D. vitifoliae* genome (v3.1; https://bipaa.genouest.org/is/) using BLASTN with the minimum identity of 90%, and transcripts having lower identity were discarded. Because RNA-Seq-based transcriptome generally includes splicing variants and truncated transcripts of same genes, the transcripts were merged to genome loci, and gene annotation was then conducted on APOLLO, a web-based genome annotation browser that integrated the genome sequence [41], automated gene models (OGS3.2), and 10 datasets of RNA-Seq reads spanning several populations in France and North America, whole body, egg, and feeding life stages, and native and cultivated hosts of origin. While gene models were predicted at most loci, it was difficult or even impossible at others that lacked automated gene models and had poor RNA-Seq support or severe intragenic sequence gaps, which were therefore discarded. The protein sequences of the annotated gene models were subsequently retrieved, among which, the full-length or complete ones were retained for further analysis, while the incomplete ones with truncated terminus were aligned to their corresponding transcript-derived protein sequences using BLASTP, and the terminal gaps were filled using the latter with identities ≥99%, if available. Here, the criteria of determining full-length sequences were: 1) the presence of a conserved start codon (ATG) and a stop codon (TAA, TAG, or TGA) at the beginning and the end of the coding region, respectively; 2) the absence of other start codon upstream of the predicted start codon within the gene sequence; and 3) similarity to a predicted full-length paralog, if available. The rescued sequences, pooled together with the earlier retrieved full-length sequences, were further examined using HMMERSCAN to ensure the presence of a RING domain (Fig. 1a). The collected RING protein genes were named based on their genomic location in the form of ‘RING-X-Y’ where ‘X’ and ‘Y’ indicated the genome scaffold number and approximate nucleotide position, respectively.

### Determination of putative secretory and non-secretory RING proteins

Secretory function of RING proteins was performed (Fig. 1b) using a pipeline adopted from [24]. The identified *D. vitifoliae* RING finger proteins were firstly examined for the presence of N-terminal secretory signal peptide using SignalP (v3.0, v4.1 and v5.0 [42]). If no signal peptide was detected by SignalP, the proteins were considered as non-secretory. The remaining proteins containing predicted signal peptide were subsequently processed for transmembrane domain prediction using TMHMM (v2.0). Proteins with predicted transmembrane domains were considered as non-secretory and therefore pooled together with earlier identified signal peptide-absent proteins. Those without detected transmembrane domains were considered as secretory and named secretory proteins with RING function (SPRINGs [43]).

### Bioinformatics analyses of RING finger proteins

Sequences of *D. vitifoliae* non-secretory RINGs and SPRINGs were retrieved to search against a preformatted NCBI database including the non-redundant (NR) protein database and the Refseq database updated until 01/29/2019 using BLASTP (e-value ≤1e-3). The top hit e-value and species, if available, of each query protein were used to compare the genetic conservation between the secretory and non-secretory proteins. For query sequences not having a BLAST hit, the threshold ‘1e-3’ was used as their e-values. The logarithm to base 10 of each e-value (the value of 0 was substituted by 1e-180 because log (0) is undefined) was calculated and converted to a positive number. Estimation of the nonsynonymous and synonymous substitution rate ratio (dN/dS) was conducted in the secretory and non-secretory RING protein groups, respectively. First, the reciprocal BLAST hit (RBH) pairs were determined using BLASTP (v2.2.30+) searches, and the codon-based sequence alignment of each RBH pair was performed using MAFFT (v7.427). The poor-aligned regions were trimmed using GBLOCKS (v0.91b), and the pairwise dN and dS values were calculated to convert to dN/dS ratio using CODEML in the PAML package (v1.7.3).

RNA-Seq-based expression analysis was performed by combining raw reads from leaf-feeding adults (*n* = 2), and eggs (*n* = 1) from [38] with leaf feeding adults (*n* = 3) and egg (*n* = 2) samples from TN, collected and processed as described above. RNA-Seq reads were assessed for quality (FastQC; http://www.bioinformatics.babraham.ac.uk/projects/fastqc/ [44];) and edited using BBDuk (sourceforge.net/projects/bbmap/). Remaining adapter artifacts were trimmed from the right with k = 23, mink = 10, hdist = 1. Low quality sequences were trimmed from the right or left of base calls with a Phred score lower than 20. Short reads smaller than 20 bases after trimming were discarded. Reads were mapped to the annotated reference genome (v3.2) using hisat2.1.0 with a k value of 5, minimum intron length of 30 bases, and maximum intron length of 3000 bases. Counts were obtained using functions in packages BiocParallel, GenomicFeatures, and Rsamtools as outlined by SystemPipeR [45] against the genome reference. For mapped genes, counts totaling ≤2 were excluded, and remaining genes were compared for differential gene expression using EdgeR-limma [46] with the LM y~ 0 + trt to compare all treatment combinations. Contrasts of interests were defined as stage = mean adult samples (*n* = 5) – mean egg samples (*n* = 3) and location = mean TN samples (*n* = 5) – mean France samples (*n* = 3). Genes were determined significant at the adjusted *P*. value (FDR) < 0.1 and logFC > 1 or < − 1 (fold change > 2 or < − 2). Although SPRINGs and RING domain containing genes were the target of the comparative analyses, we also present expression profiles for egg-specific proteins and general mitochondrial genes as positive and negative controls. Gene ontologies for these controls were determined by searching annotated genes in the genome for “eggshell” and “mitochondrial”.

### Phylogeny of RING finger proteins

Phylogenetic analyses were performed on the putatively secretory and non-secretory RING finger proteins, respectively. Amino acid sequences were first aligned using MAFFT (v7.427) and the poorly aligned regions were trimmed using TRIMAL (v1.4.1) based on a gap threshold of 0.25. The best-fit models of protein evolution were determined using ProtTest (v3.4.2), which were FLU+G + F for the secretory proteins and WAG+G + F for the non-secretory proteins, according to Bayesian information criterion. Because the FLU protein substitution matrix is not available in the phylogenetics program MRBAYES, the second best-fit model, JTT + G + F, was used instead for the phylogenetic analysis of the secretory RING proteins. We ran the analyses using two runs with 4 chains per run in MRBAYES (v3.2.6) until the standard deviation of split frequencies (SDOSF) between runs dropped below 0.05 or near the lowest value. Specifically, the secretory proteins were run for 1.4 million generations to drop SDOSF below 0.05 and the non-secretory proteins were run for 2.8 million generations to drop SDOSF to 0.07, the lowest within 5 million generations of running. The first 25% of generations were then discarded and the remaining generations were used to build a 50 majority-rule consensus tree.

### Yeast two-hybrid assay

Fresh leaves of *Vitis vinifera* were collected for total RNA extraction using the RNeasy Plant Mini Kit (Qiagen), and the yeast two-hybrid prey library was constructed using the Make Your Own “Mate & Plate” Library System (Clontech) following the manual provided. A secretory RING protein gene, RING-16-700228, was selected as bait to perform the yeast two-hybrid assay using the Matchmaker Gold Yeast Two-Hybrid System and the instruction supplied (Clontech). First, the bait gene, with the signal peptide-encoding sequence removed, was cloned into plasmid pGBDT7, and the insert was confirmed by Sanger sequencing. The bait plasmid construct was then transformed into Y2HGold yeast strain and the auto-activation test was conducted to ensure it did not autonomously activate the reporter genes in the absence of a prey protein. The bait clone was pooled and incubated with plant prey cDNA library for 24 h to allow mating, and the diploid cells expressing interacting proteins were preliminarily screened using the Double dropout (DDO) medium, SD/−Leu/−Trp, supplemented with X-a-Gal and Aureobasidin A (DDOXA). These clones were further screened on the higher stringency selective medium, the Quadruple dropout (QDO) medium, SD/−Leu/−Trp/−Ade/−His, supplemented with X-a-Gal and Aureobasidin A (QDOXA). The prey clones that interacted with the bait determined by QDOXA screening were retrieved for pairwise yeast two-hybrid assay. Briefly, the prey plasmids rescued from yeast cells were respectively co-transformed with bait plasmids into Y2HGold yeast cells. The presence of both bait and prey plasmids in yeast cells was selected using DDO medium, and the protein-protein interactions were tested on QDOXA medium with proper control included.

### Transcriptome analysis of proteasome activity during gall formation

To better understand the proteasomal regulation in grape during native interactions with *D. vitifoliae*, we downloaded a recent RNA-Seq dataset (GEO:GSE118569 [47];) that assessed *V. riparia* response across gallicole developmental stages (sensu [48]), and reassessed differential gene expression to isolate proteasome-specific patterns. All samples (*n* = 3 for each tissue type) were used in the reanalysis. Raw reads were assessed for quality and processed as described above except the *V. vinifera* genome (Vvinifera_145_Genoscope.12X) was used as the genome reference. Reads ranged from 85 to 90% alignment, a high rate of alignment given the samples are *V. riparia* compared to a *V. vinifera* genome. Mapped genes were assessed as above except. Contrasts of interests were defined as: Gall – Leaf = mean gall samples – mean leaf samples, Leaf Development = large leaf – small leaf, Bud – Leaf = bud – mean leaf, Flower – Leaf = flower – mean leaf, Gall1 = gall1 – small leaf, Gall2 = gall2 – small leaf, Gall3 = gall3 – medium leaf, and Gall4 = gall4 – large leaf. Genes were determined significant at the adjusted P. value (FDR) < 0.1 and logFC ≥0.6 or ≤ − 0.6 (fold change ≥1.5 or ≤ − 1.5). Gene ontologies were assigned using mapping bins in MapMan (https://mapman.gabipd.org) and enrichment was determined using a Fisher’s exact test by comparing the number of differentially expressed (DE) genes relative to controls within proteasome-related bins to the total number of DE genes for each tissue type (each gall stage, buds and flowers), and across development of the gall and leaf. To compare this analysis to the original published transcriptome, we evaluated several key genes found differentially expressed or not in galled versus non-galled leaf tissue in the original dataset (Additional file 1).

## Additional file

**Additional file 1: Table S1.** Differential expression profiles for RING domain containing genes for each contrast: Stage, Location. **Table S2.** Numbers of up (down) regulated genes for each process defined within the UPS and whether the specific contrast showed enrichment relative to all other differentially expresssed genes.

## Data Availability

The datasets supporting the conclusions of this article are included within the article (and its additional file) and in the NCBI BioProject/SRA: PRJNA552348 (https://www.ncbi.nlm.nih.gov/bioproject/552348), PRJNA561603 (https://www.ncbi.nlm.nih.gov/bioproject/561603).

## Abbreviations

A, C, G, T: Adenine, cytosine, guanine, thymine
aa: Amino acid
CSLD5: Cellulose synthase-like protein 5
DE: Differentially expressed
HECT: Homology to E6AP C-terminus
JA: Jasmonic acid
ORF: Open reading frame
RING: Really interesting new gene
RPS4: Ribosomal protein S4
SA: Salicylic acid
SPRING: Secretory protein with RING domain
UPS: Ubiquitin-proteasome system
Y2H: Yeast-two hybrid
